# Heterogeneity of Aging: Implications for Team Care and Team Science

**DOI:** 10.1093/geroni/igaa057.3144

**Published:** 2020-12-16

**Authors:** George Kuchel, Richard Fortinsky, Luigi Ferrucci

## Abstract

Increasing heterogeneity with aging is a deeply held belief in gerontology often used to combat generalizations and ageist stereotypes regarding older adults. Nevertheless, the vast majority of published studies do not report or discuss variability in their findings with aging, instead focusing on average differences between age groups. Yet, when data diversity is examined, most studies do find increased heterogeneity with aging across all domains – biological, immunological, behavioral, social, clinical, and population. Although heterogeneity has been described across the aging literature, including most GSA journals, little or no effort has been made to define and better understand the very nature of heterogeneity as a conserved feature of aging evident across all of its varied dimensions. It is well established that multidisciplinary team-based approaches are essential to clinical care of older adults, to research efforts in aging, and to the training of future generations of scientists, clinicians, educators and others in the aging field. Over the last 75 years, GSA has been a leading and unique vehicle for the development of multidisciplinary and interdisciplinary dialogue and collaborations involving its six membership sections. This symposium will provide a unique opportunity to begin a multidisciplinary dialogue designed to engage the broader GSA community in determining shared, as well as distinct, features of heterogeneity as they are manifested in terms of biology, immunology, behavioral and social considerations, and clinical and population issues, with ultimate impact on health policy and practice.

